# Supplementary material for: An Insight into Symmetrical Cyanine Dyes as Promising Selective Antiproliferative Agents in Caco-2 Colorectal Cancer Cells
Source: Molecules. 2022 Sep 7;27(18):5779. doi: 10.3390/molecules27185779 (PMC9503608; doi:10.3390/molecules27185779)
Supplement: Supplementary file 1 [file molecules-27-05779-s001.zip › molecules-1888269-supplementary.pdf]

Supporting Information

An Insight into Symmetrical Cyanine Dyes as Promising Selective Antiproliferative Agents in Caco-2 Colorectal Cancer Cells

João L. Serrano 1,2,*,†, Ana Maia 1,2,†, Adriana O. Santos 1, Eurico Lima 1,3, Lucinda V. Reis 3, Maria J. Nunes 2, Renato E. F. Boto 1,2, Samuel Silvestre 1,2,4 and Paulo Almeida 1,2,*

1 CICS-UBI-Health Sciences Research Center, University of Beira Interior, Av. Infante D. Henrique, 6201-506 Covilhã, Portugal
2 Department of Chemistry, University of Beira Interior, Rua Marquês de Ávila e Bolama, 6201-001 Covilhã, Portugal
3 CQVR-Chemistry Centre of Vila Real, University of Trás-os-Montes and Alto Douro, Quinta de Prados, 5001-801 Vila Real, Portugal
4 CNC-Center for Neuroscience and Cell Biology, University of Coimbra, Rua Larga, 3004-517 Coimbra, Portugal
* Correspondence: serrano.joao@ubi.pt (J.L.S.); paulo.almeida@ubi.pt (P.A.)
† These authors contributed equally to this work.

Table of Contents

Table S1 – Characteristic ^{1}H and ^{13}C NMR spectra signal of dyes **2-19**. 3

Table S2 – Data for in vitro effects of bisbenzothiazole **1**, dyes **2-19** and 5-fluorouracil (5-FU) on cell viability of human adenocarcinoma cell lines of the prostate (PC-3), breast (MCF-7) and colorectal (Caco-2), and non-tumour cell line of normal human dermal fibroblasts (NHDF), after 72 h of incubation at the single concentration of 10 μM.[a] 4

Figure S1 – Spectral output of the emission spectra from Red (R). Green (G). Blue (B) and White (W) lights of a RGBW LED projector (220-240 V and 30 W. Luxtar). Intensity was normalized and are present as arbitrary units (a.u.). 5

Figure S2 - In vitro photocytotoxic effects of cyanine dye **EL4** on relative cell viability of non-tumour cell line of normal human dermal fibroblasts (NHDF). Exposure to the dye **EL4** was evaluated after 72 h of incubation in the dark and after irradiation with a LED system. 5

Figure S3 – ^{1}H and ^{13}C NMR spectra of bisbenzothiazole **1**. 6

Figure S4 – ^{1}H and ^{13}C NMR spectra of monomethine cyanine dye **2**. 7

Figure S5 – ^{1}H and ^{13}C NMR spectra of monomethine cyanine dye **3**. 8

Figure S6 – ^{1}H and ^{13}C NMR spectra of monomethine cyanine dye **4**. 9

Figure S7 – ^{1}H and ^{13}C NMR spectra of monomethine cyanine dye **5**. 10

Figure S8 – ^{1}H and ^{13}C NMR spectra of monomethine cyanine dye **6**. 11

Figure S9 – ^{1}H and ^{13}C NMR spectra of monomethine cyanine dye **7**. 12

Figure S10 – ^{1}H and ^{13}C NMR spectra of monomethine cyanine dye **8**. 13

Figure S11 – ^{1}H and ^{13}C NMR spectra of monomethine cyanine dye **9**. 14

Figure S12 – ^{1}H and ^{13}C NMR spectra of monomethine cyanine dye **10**. 15

Figure S13 – ^{1}H and ^{13}C NMR spectra of monomethine cyanine dye **11**. 16

Figure S14 – ^{1}H and ^{13}C NMR spectra of monomethine cyanine dye **12**. 17

Figure S15 – ^{1}H and ^{13}C NMR spectra of monomethine cyanine dye **13**. 18

Figure S16 – ^{1}H and ^{13}C NMR spectra of monomethine cyanine dye **14**. 19

Figure S17 – ^{1}H and ^{13}C NMR spectra of trimethine cyanine dye **15**. 20

Figure S18 – ^{1}H and ^{13}C NMR spectra of trimethine cyanine dye **16**. 21

Figure S19 – ^{1}H and ^{13}C NMR spectra of heptamethine cyanine dye **17**. 22

Figure S20 – ^{1}H and ^{13}C NMR spectra of heptamethine cyanine dye **18**. 23

Figure S21 – ^{1}H and ^{13}C NMR spectra of heptamethine cyanine dye **19**. 24

Figure S22 – ESI-HRMS spectrum of monomethine cyanine dye **8**. 25

Figure S23 – ESI-HRMS spectrum of monomethine cyanine dye **9**. 26

Figure S24 – ESI-HRMS spectrum of monomethine cyanine dye **10**. 27

Figure S25 – ESI-HRMS spectrum of monomethine cyanine dye **11**. 28

Figure S26 – ESI-HRMS spectrum of monomethine cyanine dye **14**. 29

Table S1 – Characteristic ^{1}H and ^{13}C NMR spectra signal of dyes **2-19**.

Dye	^{1}H NMR		^{13}C NMR	
	C=C<u>H</u>	$N^{1/2+}C\underline{H}_n$	C=C<u>H</u>	$NC\underline{H}_n$
**2**	6.70 (s, 1H)	4.02 (s, 6H)	83.0	34.2
**3**	6.72 (s, 1H)	4.03 (s, 6H)	82.9	34.2
**4**	6.70 (s, 1H)	4.02 (s, 6H)	82.9	34.1
**5**	6.72 (s, 1H)	4.03 (s, 6H)	82.9	34.1
**6**	6.75 (s, 1H)	4.70 (q, *J* = 7.1 Hz, 4H)	81.9	41.6
**7**	6.75 (s, 1H)	4.70 (q, *J* = 7.2 Hz, 4H)	81.9	41.6
**8**	6.96 (s, 1H)	5.91 (s, 4H)	83.4	49.1
**9**	6.75 (s, 1H)	5.71 (s, 4H)	83.4	53.0
**10**	6.68 (s, 1H)	4.65 (t, *J* = 7.5 Hz, 4H)	82.5	46.2
**11**	6.68 (s, 1H)	4.65 (t, *J* = 7.4 Hz, 4H)	82.6	46.2
**12**	5.80 (s, 1H)	3.83 (s, 6H)	57.5	30.7
**13**	5.83 (s, 1H)	4.00 (s, 6H)	92.7	38.2
**14**	7.24 (s, 1H)	4.45 (t, *J* = 7.3 Hz, 4H)	96.5	54.8
**15**	8.32 (t, *J* = 13.3 Hz, 1H)<br>6.09 (d, *J* = 13.3 Hz, 2H)	4.26 (q, *J* = 7.2 Hz, 4H)	146.5<br>84.9	39.0
**16**	7.81 (t, *J* = 12.4 Hz, 1H)<br>6.79 (d, *J* = 12.4 Hz, 2H)	4.38 (q, *J* = 7.1 Hz, 4H)	151.7<br>103.2	42.3
**17**	7.78 (d, *J* = 13.4 Hz, 2H)<br>6.45 (d, *J* = 13.5 Hz, 2H)	4.39 (t, *J* = 6.8 Hz, 4H)	144.2 (C)<br>141.6 (CH)<br>125.2 (C)<br>100.0 (CH)	46.1
**18**	7.70 (d, *J* = 10.2 Hz, 4H)<br>6.60 (d, *J* = 13.2 Hz, 2H)	4.42 (t, *J* = 6.9 Hz, 4H)	144.1 (C)<br>143.1 (CH)<br>125.2 (C)<br>103.8 (CH)	46.9
**19** (IR-783)	8.26 (d, *J* = 14.1 Hz, 2H)<br>6.37 (d, *J* = 14.1 Hz, 2H)	4.22 (t, *J* = 7.4 Hz, 4H)	143.1 (C)<br>142.1 (CH)<br>125.1 (C)<br>101.8 (CH)	50.7

Table S2 – Data for i*n vitro* effects of bisbenzothiazole **1**, dyes **2-19** and 5-fluorouracil (5-FU) on cell viability of human adenocarcinoma cell lines of the prostate (PC-3), breast (MCF-7) and colorectal (Caco-2), and non-tumour cell line of normal human dermal fibroblasts (NHDF), after 72 h of incubation at the single concentration of 10 μM.[a]

	PC-3	MCF-7	Caco-2	NHDF
Control	100 ± 5.19	100 ± 13.14	100 ± 6.69	100 ± 5.45
5-FU	11.89 ± 2.1	15.99 ± 1.8	25.67 ± 1.02	12.67 ± 3.99
**1**	53.21 ± 9.68	55.73 ± 4.97	59.56 ± 5.45	31.02 ± 0.57
**2**	1.29 ± 0.24	0.85 ± 0.34	2.18 ± 0.64	5.64 ± 1.15
**3**	1.27 ± 0.31	0.52 ± 0.39	1.68 ± 0.53	3.93 ± 0.75
**4**	1.18 ± 0.36	2.26 ± 0.82	3.01 ± 0.74	3.82 ± 0.21
**5**	1 ± 0.18	1.55 ± 0.53	2.55 ± 0.58	4.42 ± 0.55
**6**	2.06 ± 0.89	1.08 ± 0.23	3.52 ± 0.3	3.37 ± 0.65
**7**	1.19 ± 0.29	0.96 ± 0.61	3.11 ± 0.94	3.34 ± 0.14
**8**	1.16 ± 0.49	1.47 ± 1.13	0.32 ± 0.03	2.04 ± 0.39
**9**	46.75 ± 14.01	48.97 ± 13.91	46.96 ± 6.81	47.91 ± 3.64
**10**	0.97 ± 0.26	0.87 ± 2.53	1.02 ± 0.42	0.1 ± 0.36
**11**	0.94 ± 0.11	0.1 ± 0.69	0.66 ± 0.44	0.15 ± 0.38
**12**	9.33 ± 4.28	10.8 ± 1.98	10.01 ± 0.93	41.01 ± 0.82
**13**	7.42 ± 1.27	11.78 ± 1.89	7.81 ± 0.51	8.37 ± 2.26
**14**	8.03 ± 2.15	22.94 ± 0.68	9.84 ± 0.93	1.99 ± 0.25
**15**	13.31 ± 1.9	6.03 ± 0.77	8.58 ± 0.92	3.72 ± 0.23
**16**	3.92 ± 0.75	1.39 ± 0.68	1.25 ± 0.39	4.53 ± 0.39
**17**	0.43 ± 0.26	11.18 ± 3.11	0.24 ± 0.17	2.26 ± 0.1
**18**	28.43 ± 7.73	51.76 ± 10.12	47.24 ± 5.29	36.59 ± 1.75
**19**	51.07 ± 5.32	94.41 ± 7.99	57.35 ± 1.75	42.01 ± 1.76

[a] Results are expressed as average values of cell viability percentage in relation with control ± standard deviation of two independent assays performed in quadruplicate. A $p < 0.001$ versus negative control in the statistical significance analysis (Student's t-test) was observed for all compounds, except for dye **19** in the MCF-7 cell line (absence of any statistical difference).

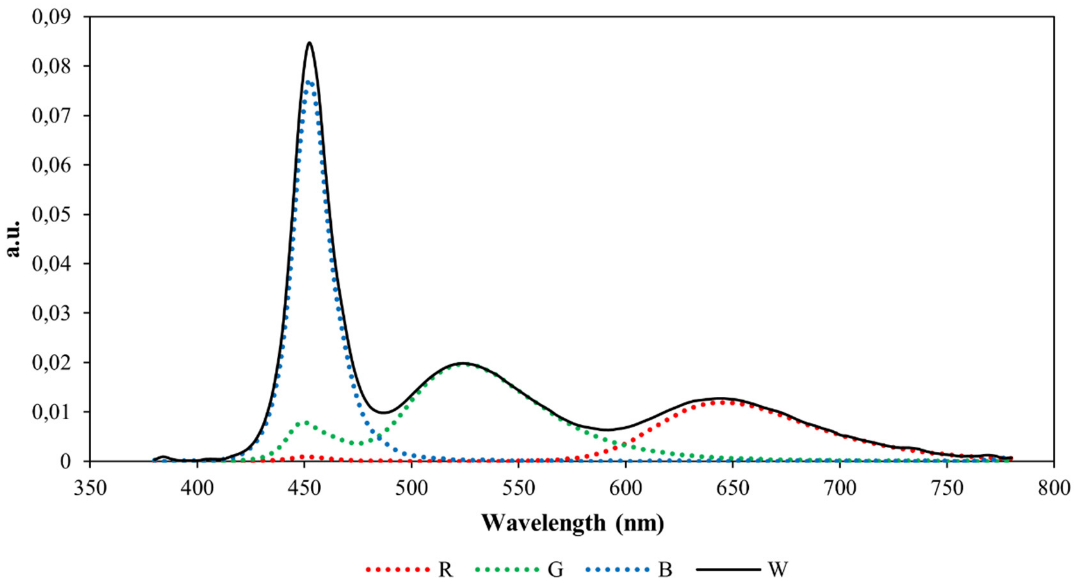

Figure S1 – Spectral output of the emission spectra from Red (R). Green (G). Blue (B) and White (W) lights of a RGBW LED projector (220-240 V and 30 W. Luxtar). Intensity was normalized and are present as arbitrary units (a.u.).

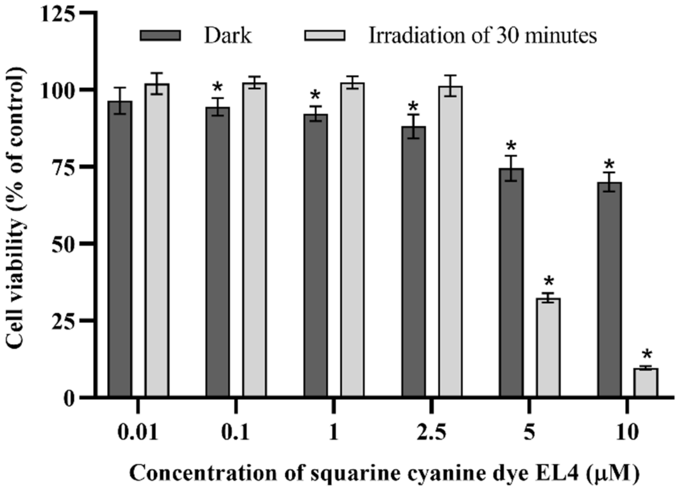

Figure S2 - *In vitro* photocytotoxic effects of cyanine dye **EL4** on relative cell viability of non-tumour cell line of normal human dermal fibroblasts (NHDF). Exposure to the dye **EL4** was evaluated after 72 h of incubation in the dark and after irradiation with a LED system. For this assay. NHDF cells were incubated 48 h with the dye **EL4** at different concentrations and then exposed to white light from a 30 W RGB LED system for 30 minutes. Cell viability was evaluated 24 h after irradiation by the MTT assay. Results are expressed as average values ± standard deviation of two independent assays performed in quadrupled. A $p < 0.05$ *versus* negative control in the statistical significance analysis (Student's t-test) was observed for the treatments evidenced by a signal (*).

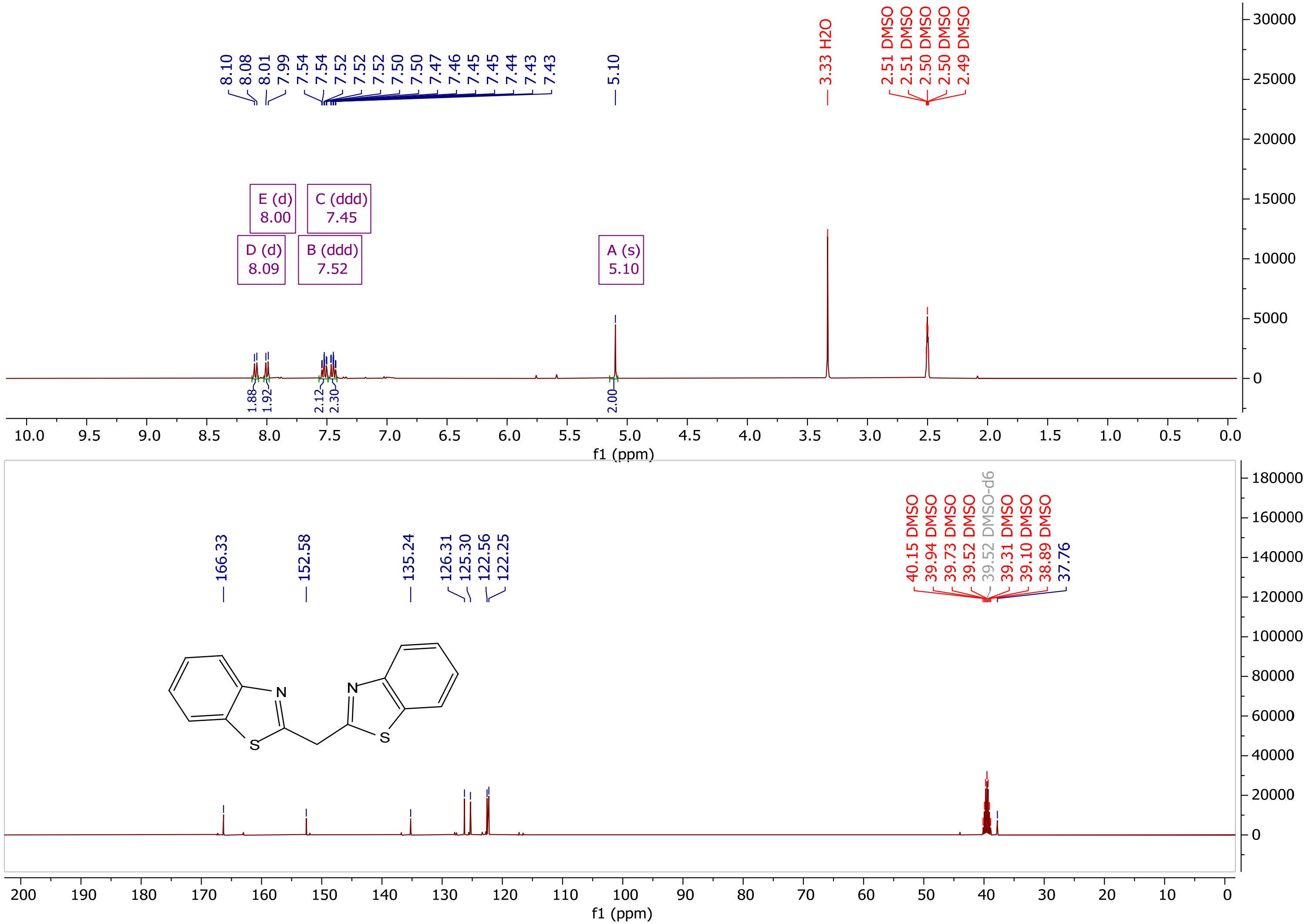

Figure S3 – ^{1}H and ^{13}C NMR spectra of bisbenzothiazole **1**.

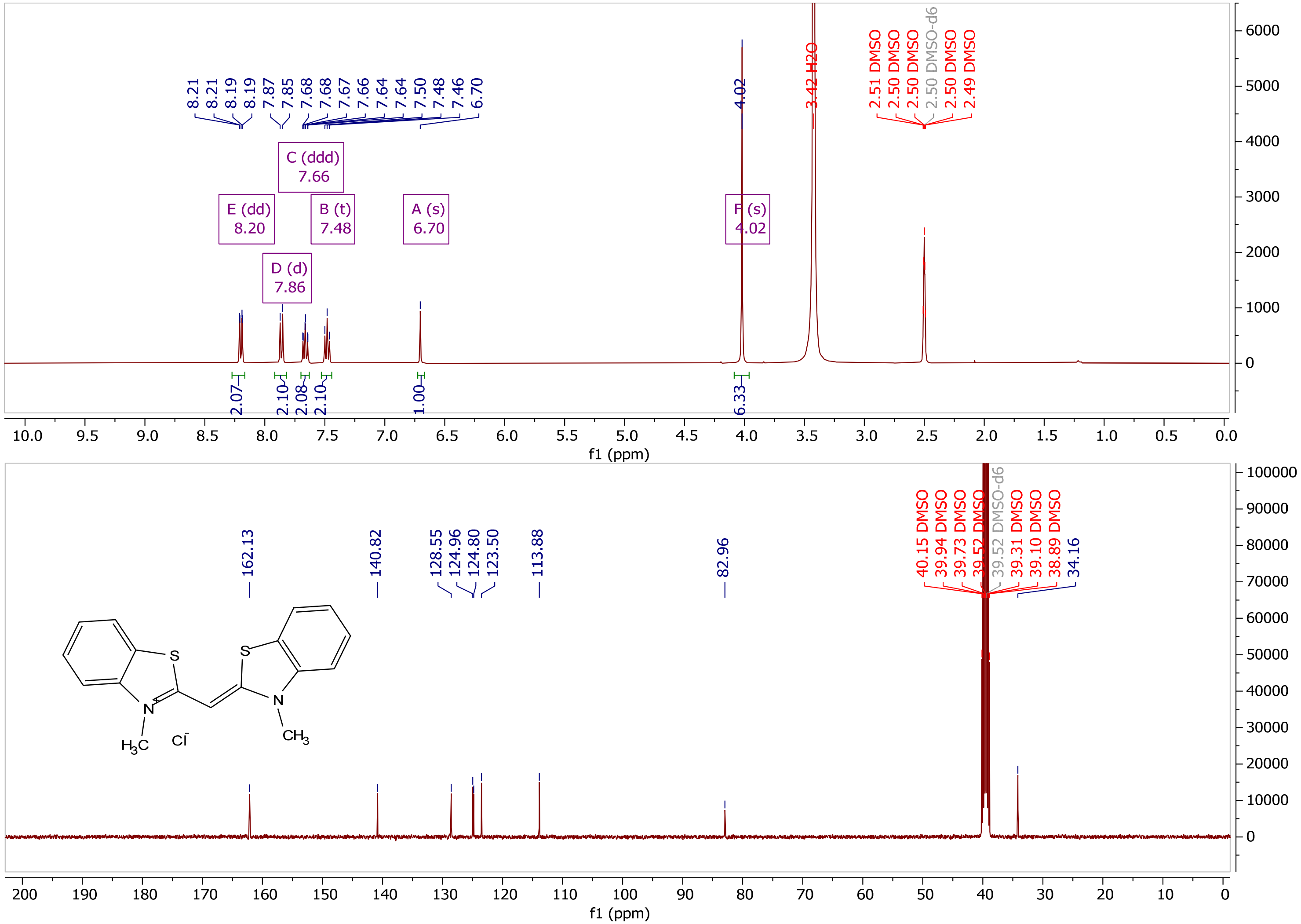

Figure S4 – ^{1}H and ^{13}C NMR spectra of monomethine cyanine dye **2**.

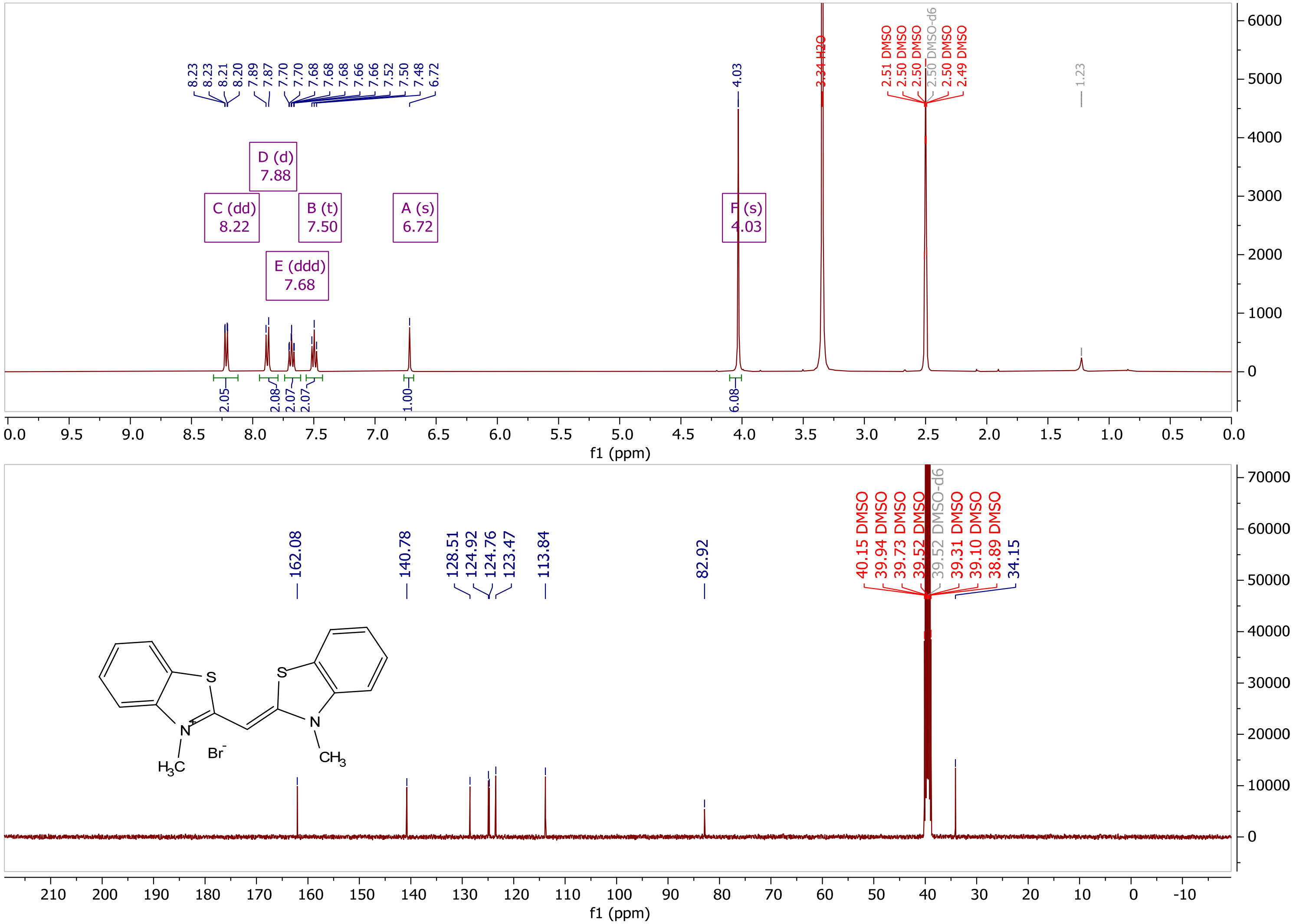

Figure S5 – ^{1}H and ^{13}C NMR spectra of monomethine cyanine dye **3**.

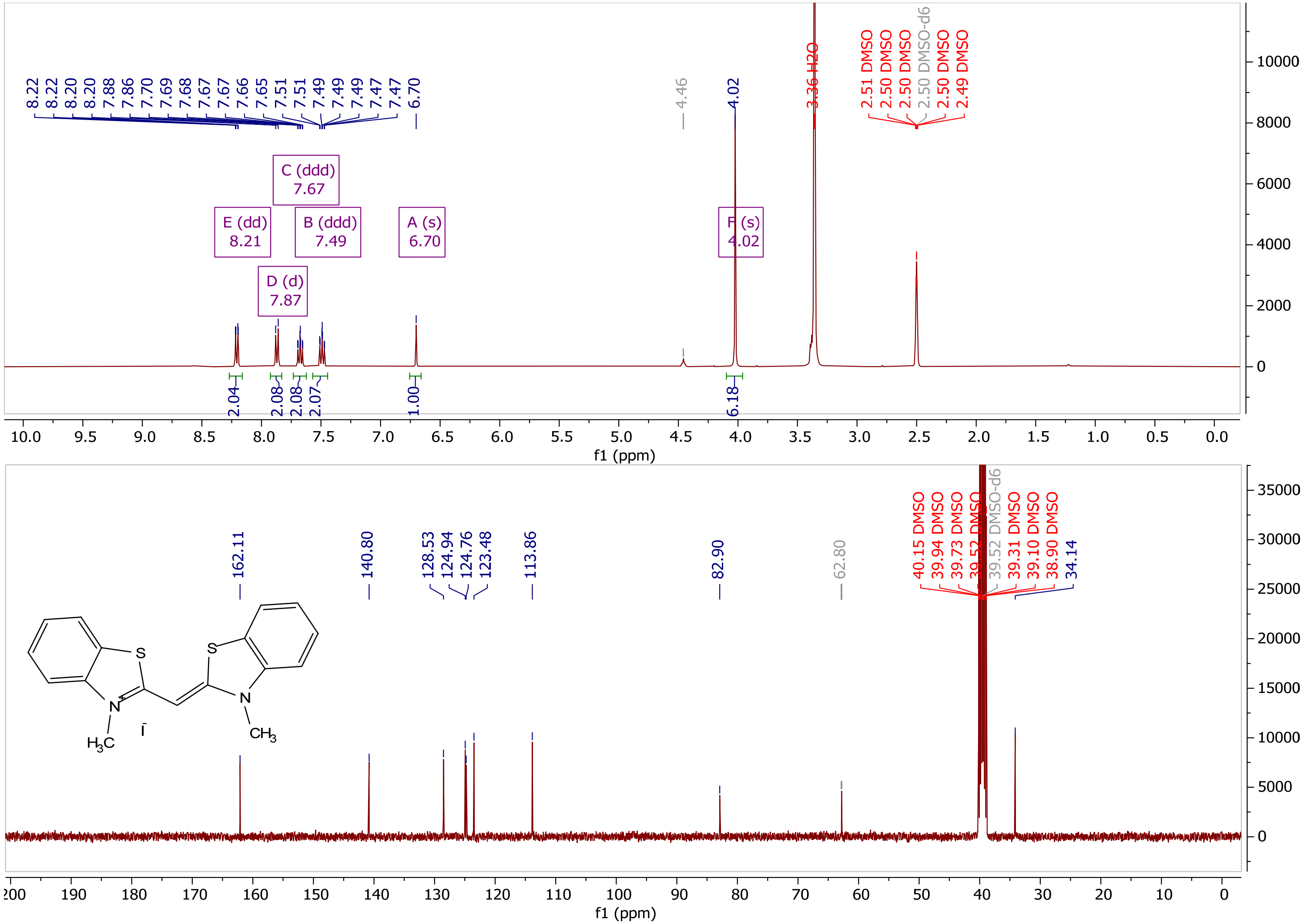

Figure S6 – ^{1}H and ^{13}C NMR spectra of monomethine cyanine dye **4**.

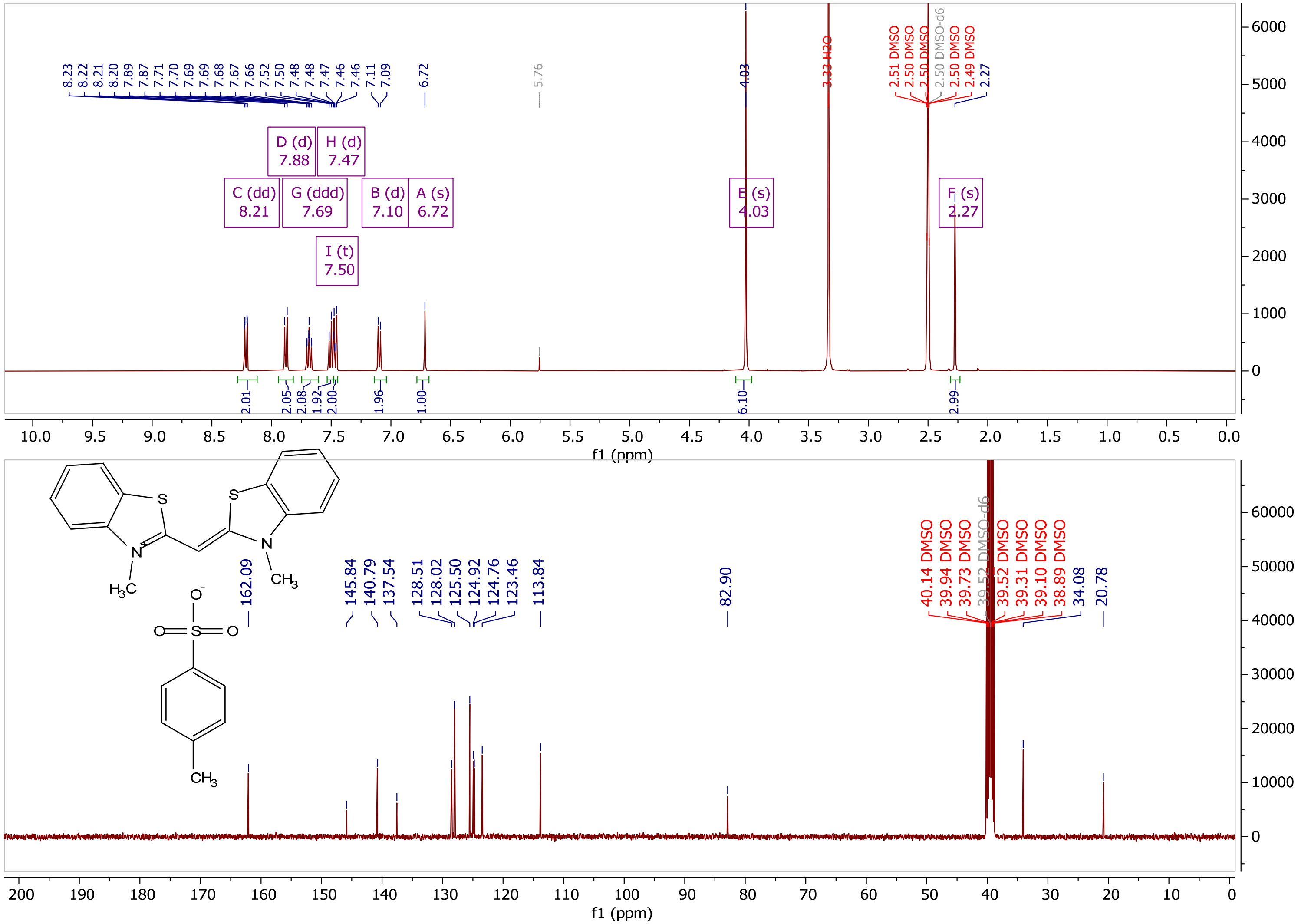

Figure S7 – ^{1}H and ^{13}C NMR spectra of monomethine cyanine dye **5**.

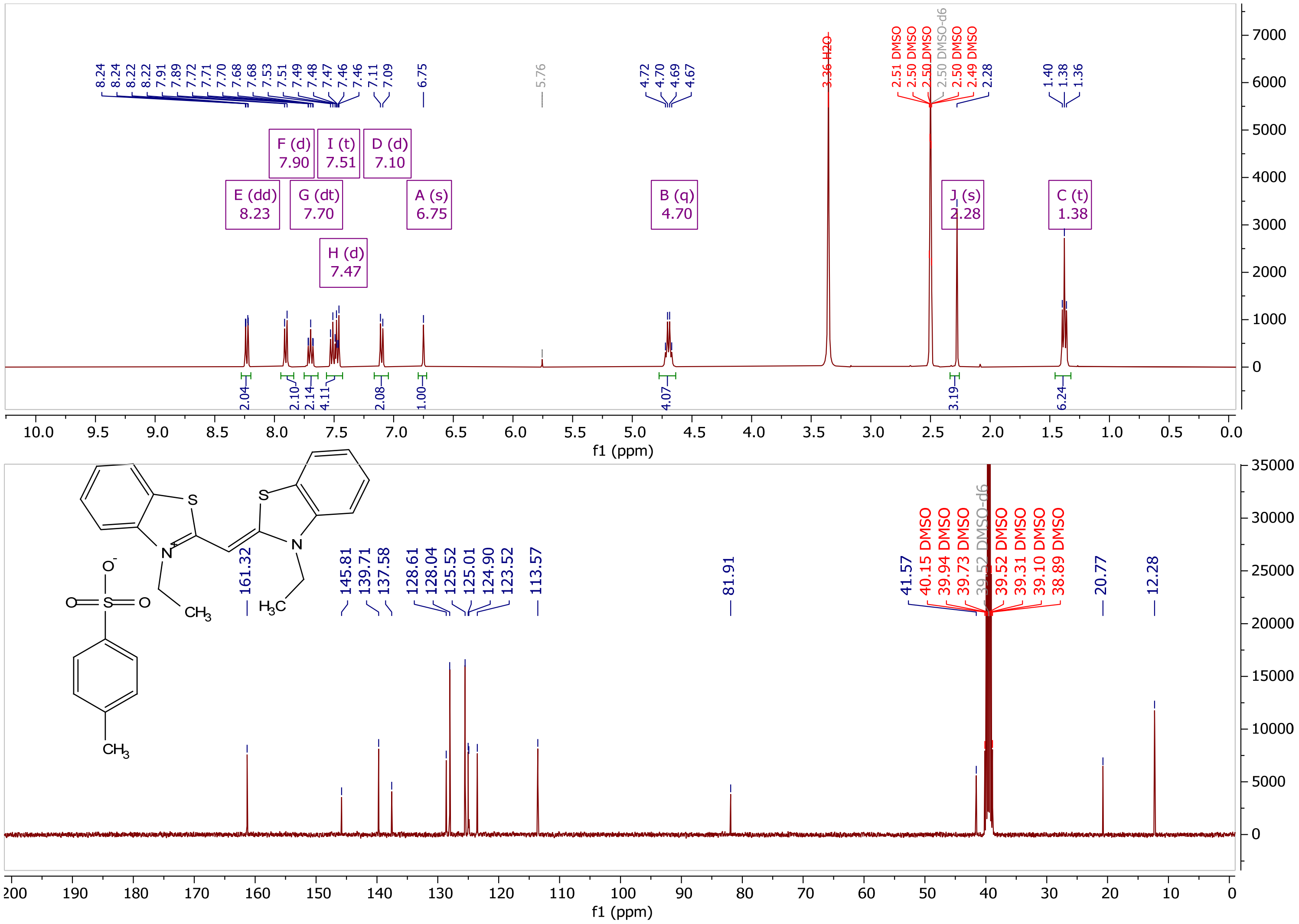

Figure S8 – ^{1}H and ^{13}C NMR spectra of monomethine cyanine dye **6**.

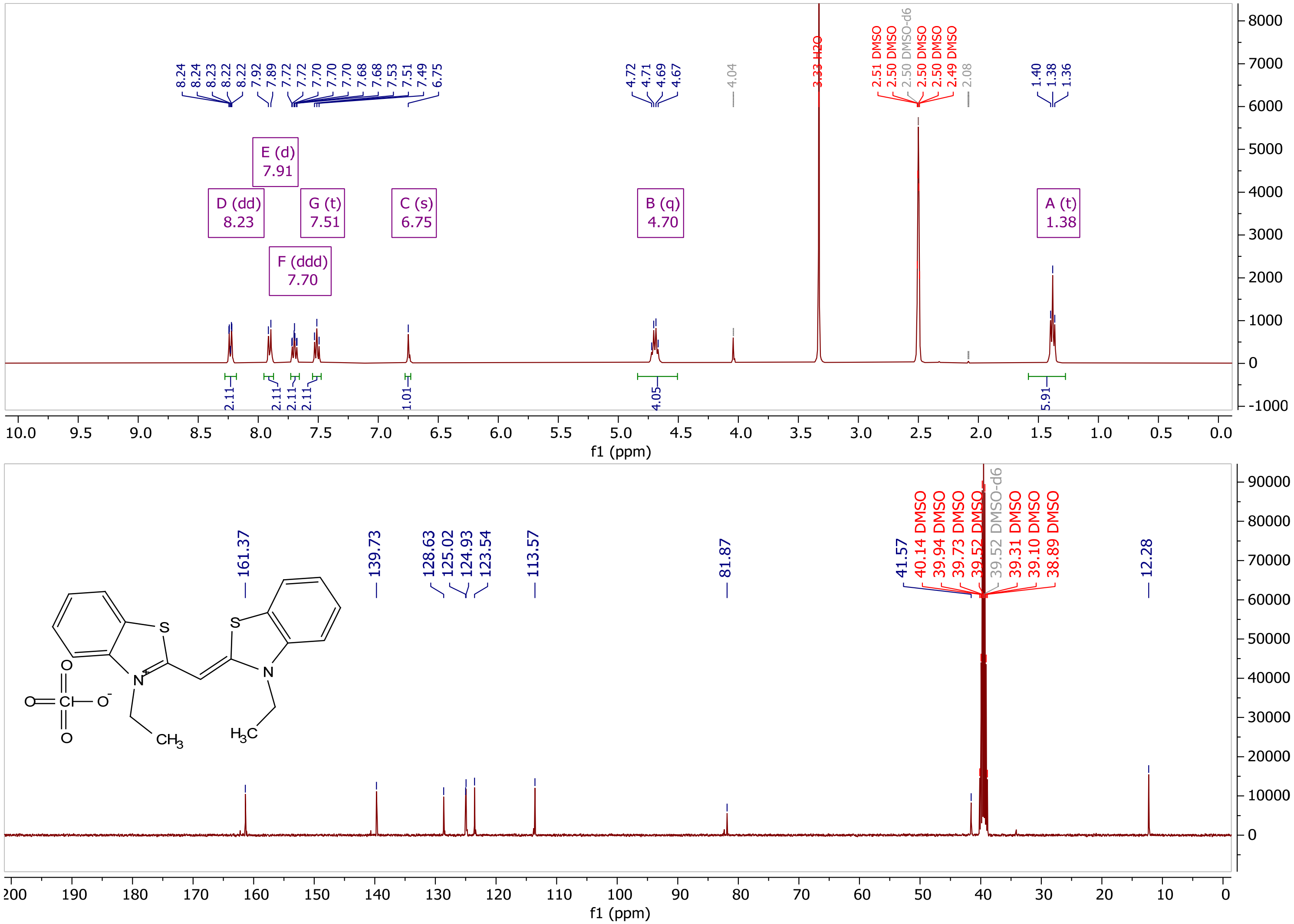

Figure S9 – ^{1}H and ^{13}C NMR spectra of monomethine cyanine dye **7**.

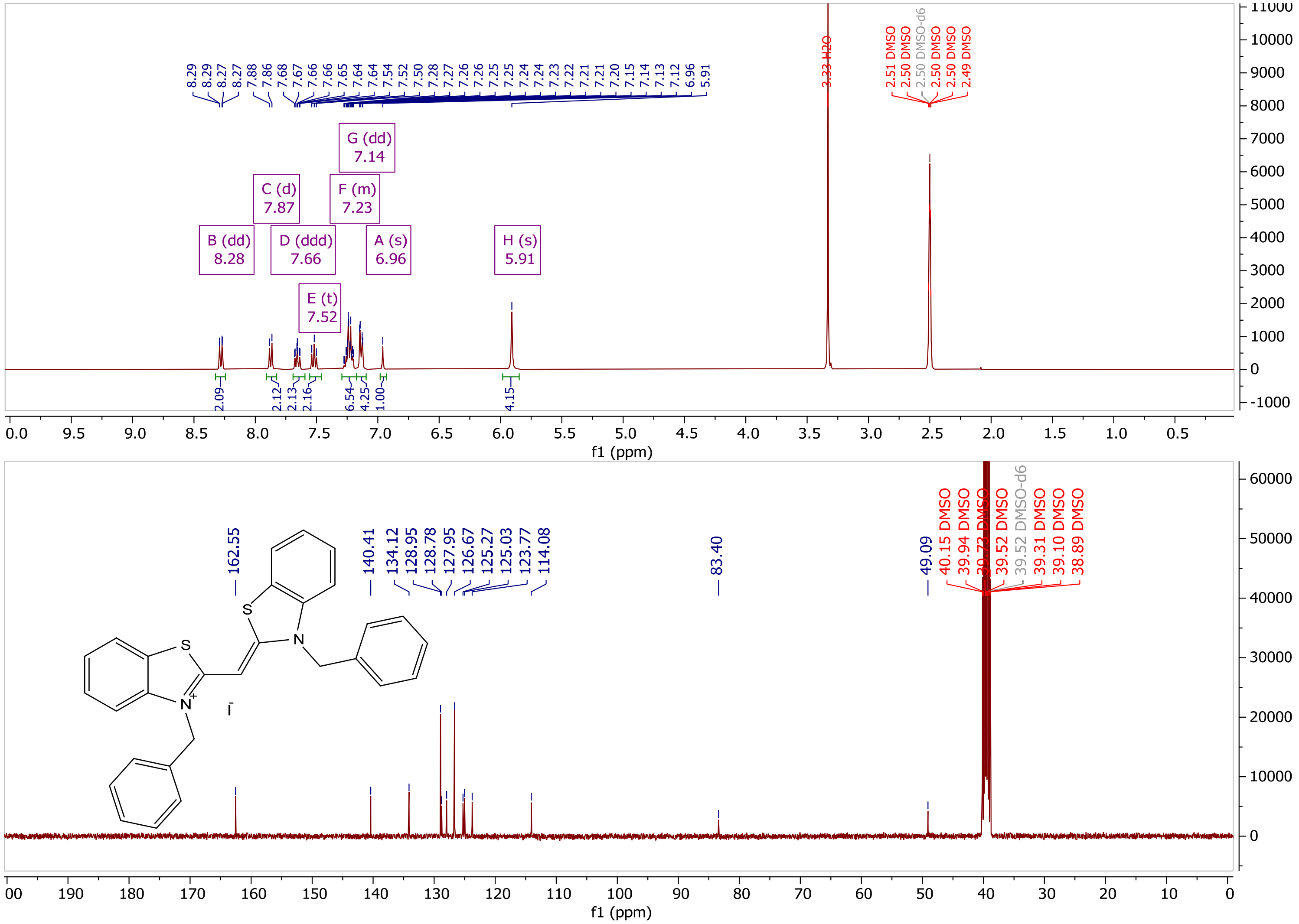

Figure S10 – ^{1}H and ^{13}C NMR spectra of monomethine cyanine dye **8**.

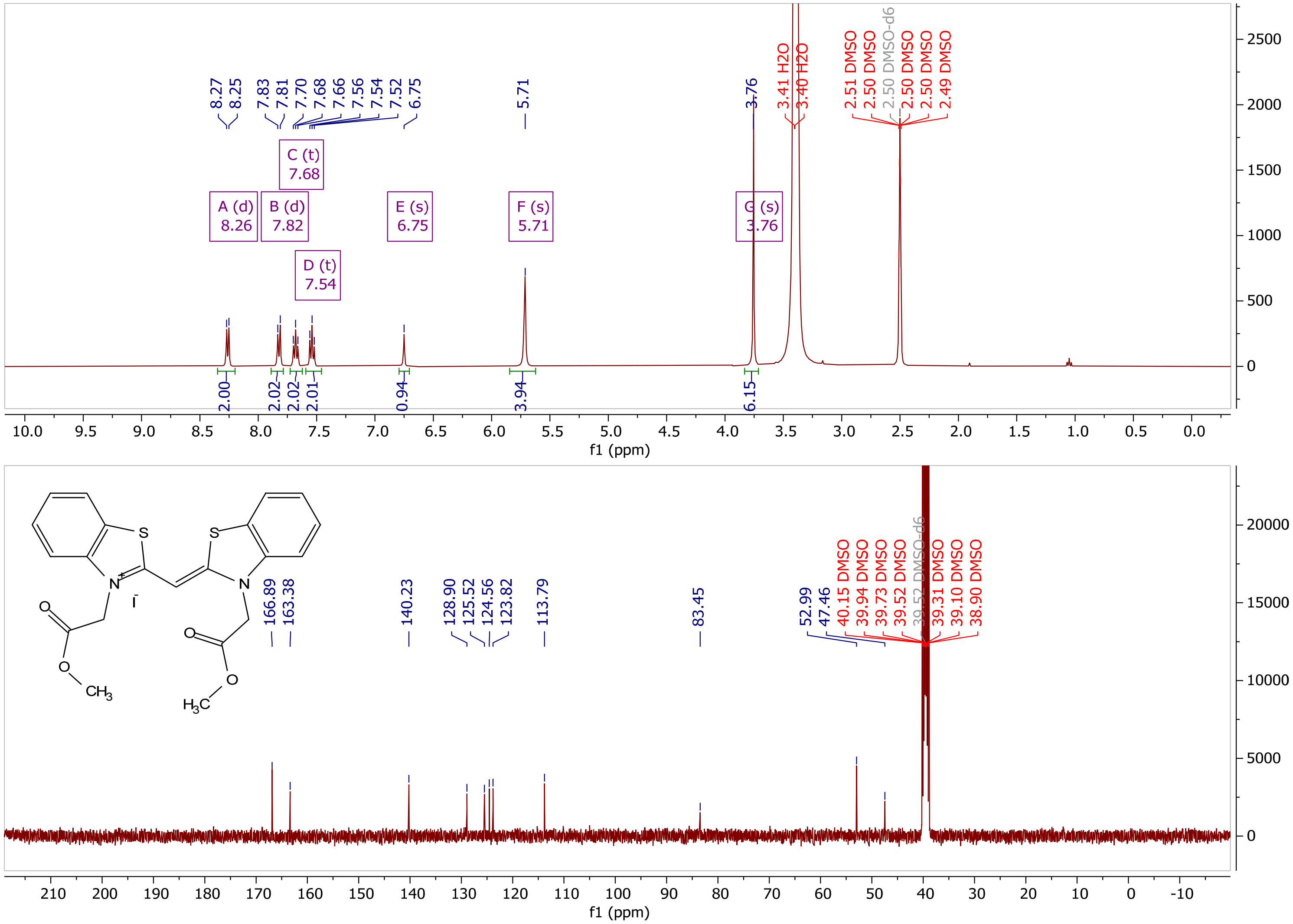

Figure S11 – ^{1}H and ^{13}C NMR spectra of monomethine cyanine dye **9**.

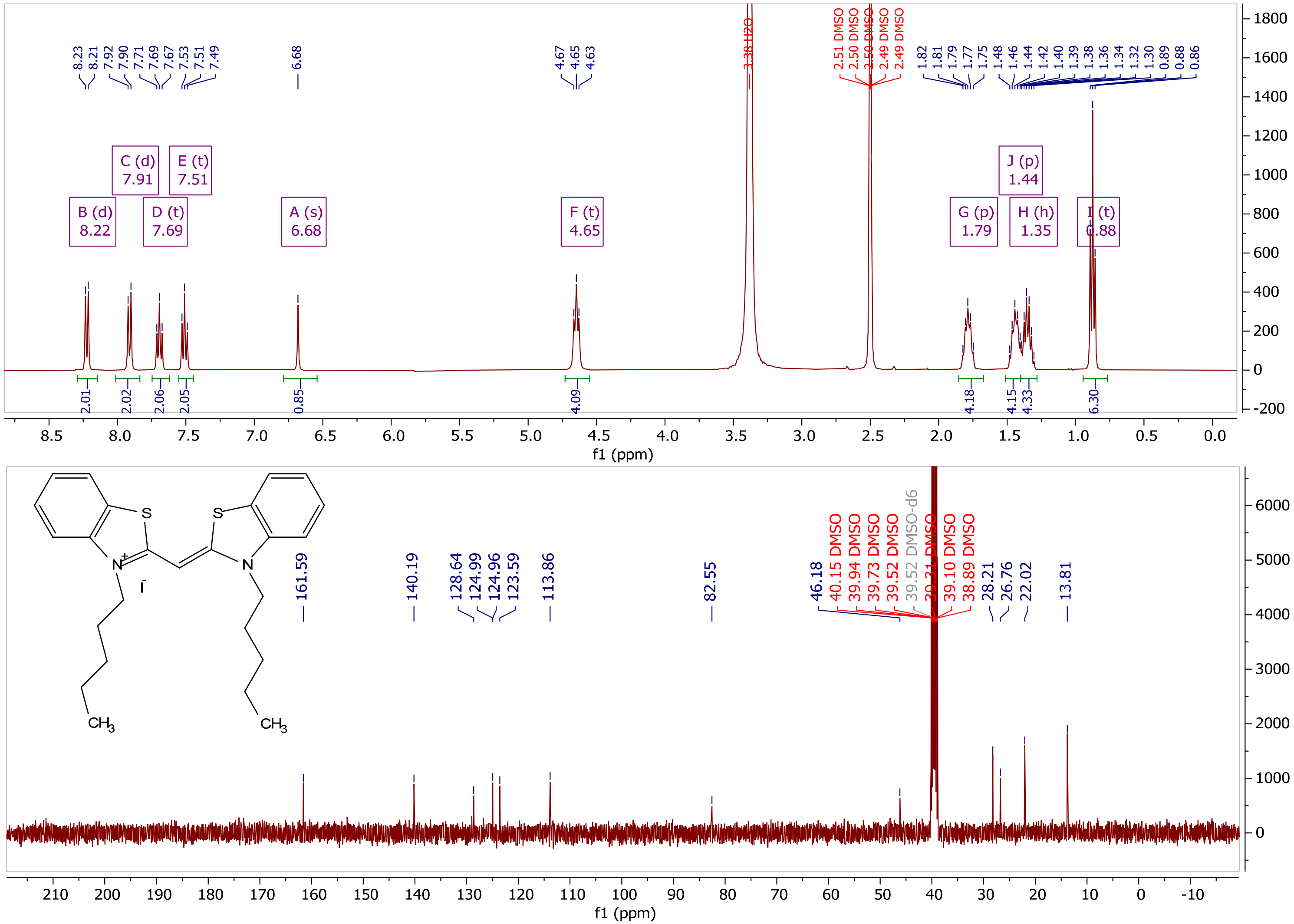

Figure S12 – ^{1}H and ^{13}C NMR spectra of monomethine cyanine dye **10**.

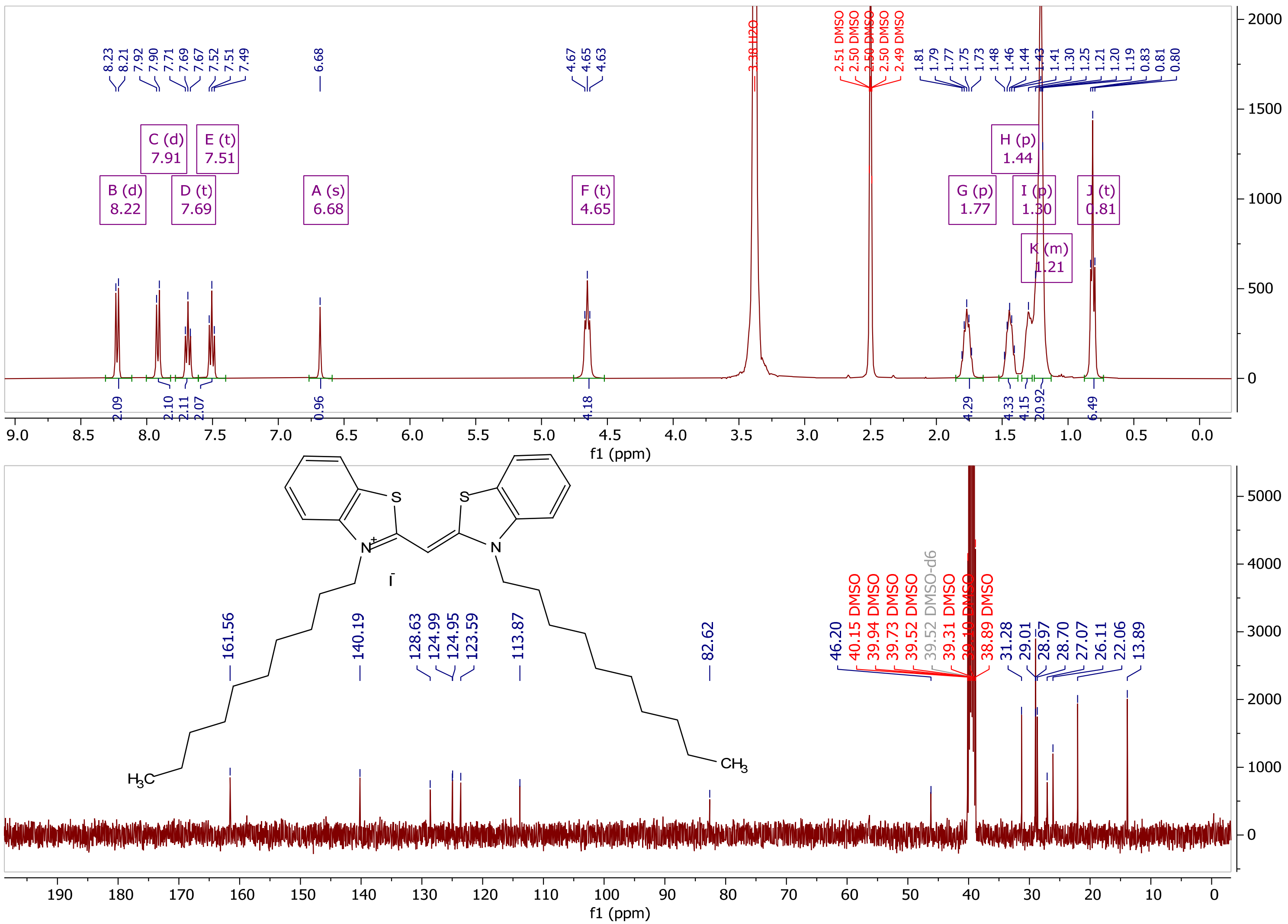

Figure S13 – ^{1}H and ^{13}C NMR spectra of monomethine cyanine dye **11**.

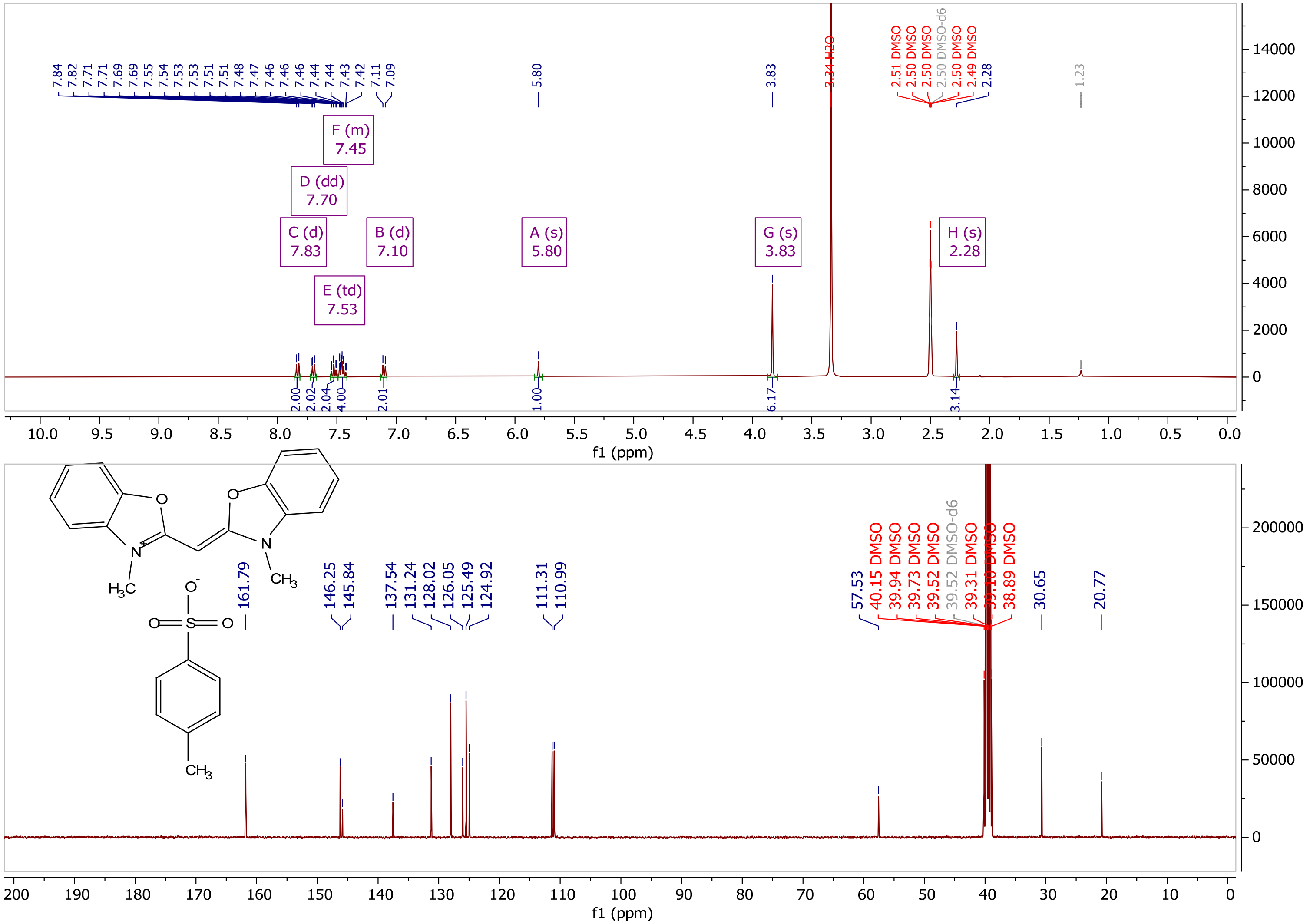

Figure S14 – ^{1}H and ^{13}C NMR spectra of monomethine cyanine dye **12**.

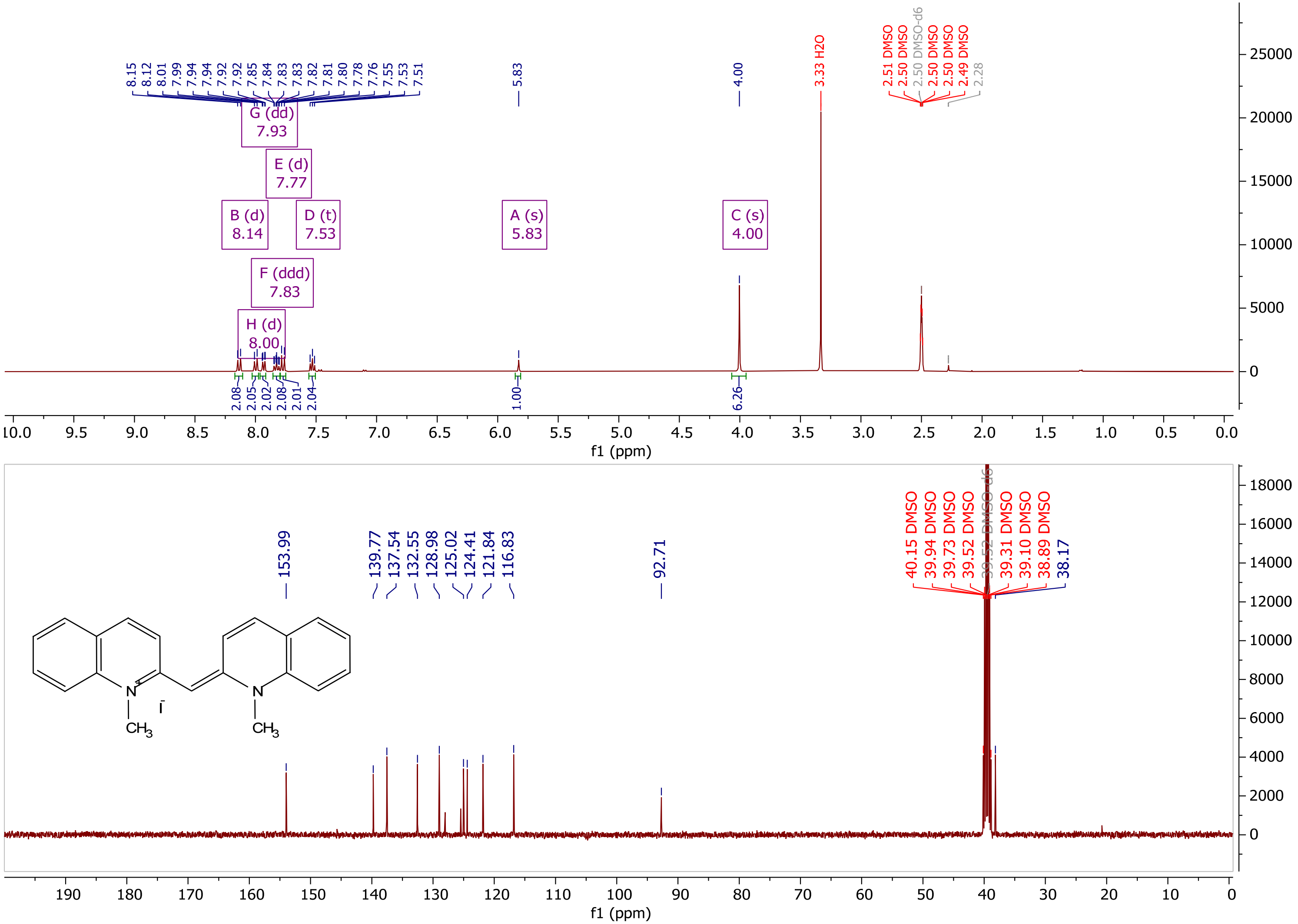

Figure S15 – ^{1}H and ^{13}C NMR spectra of monomethine cyanine dye **13**.

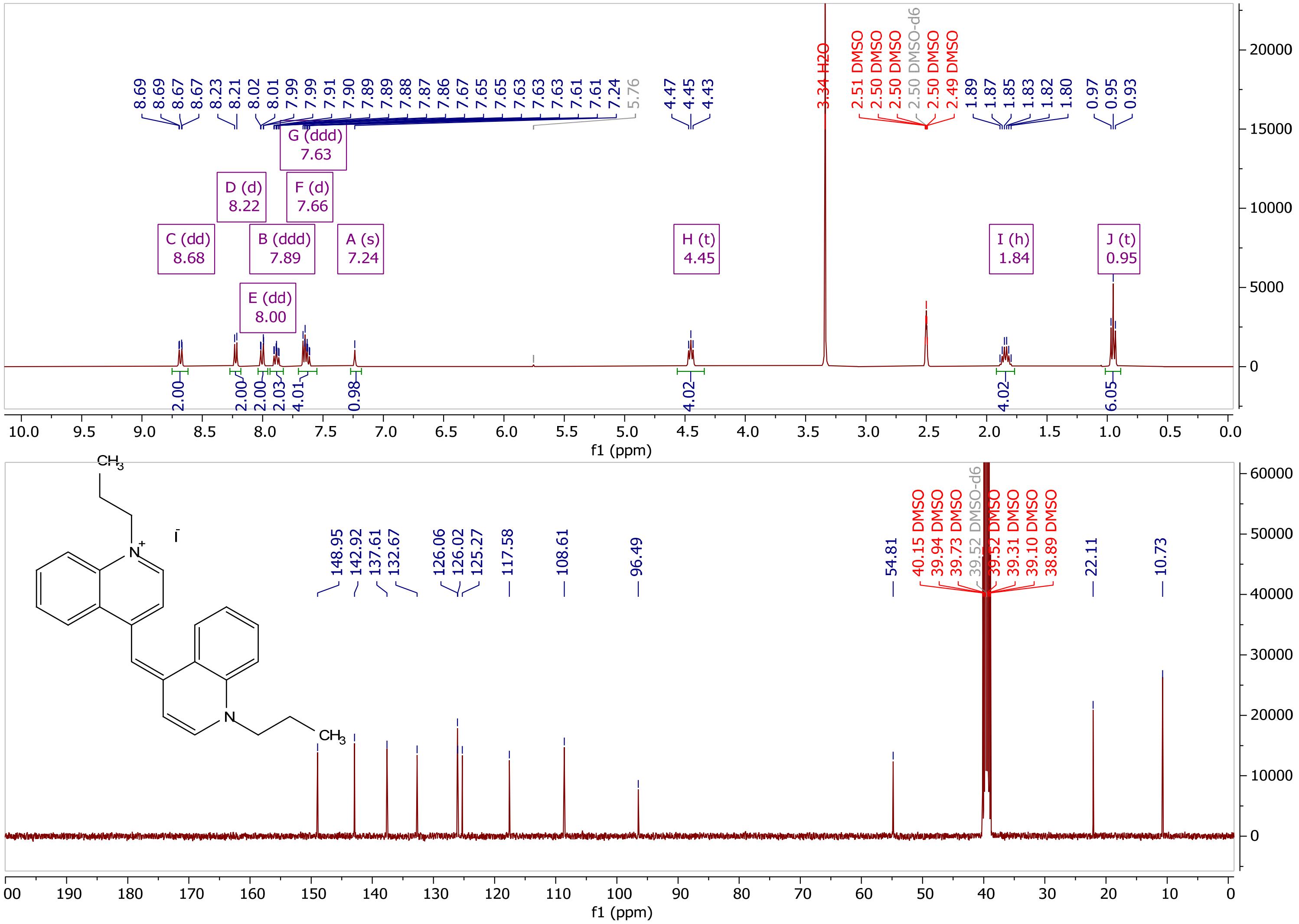

Figure S16 – ^{1}H and ^{13}C NMR spectra of monomethine cyanine dye **14**.

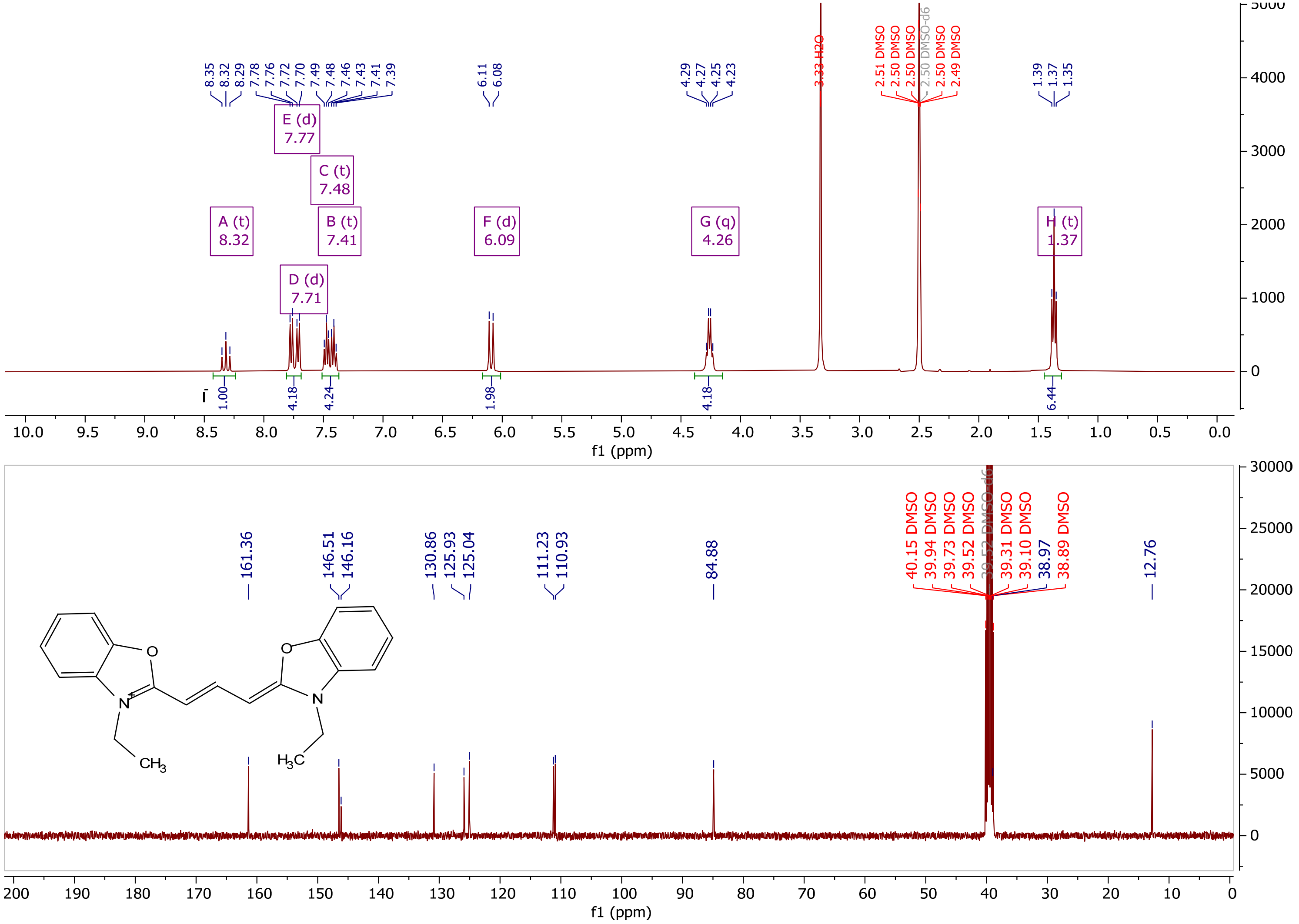

Figure S17 – ^{1}H and ^{13}C NMR spectra of trimethine cyanine dye **15**.

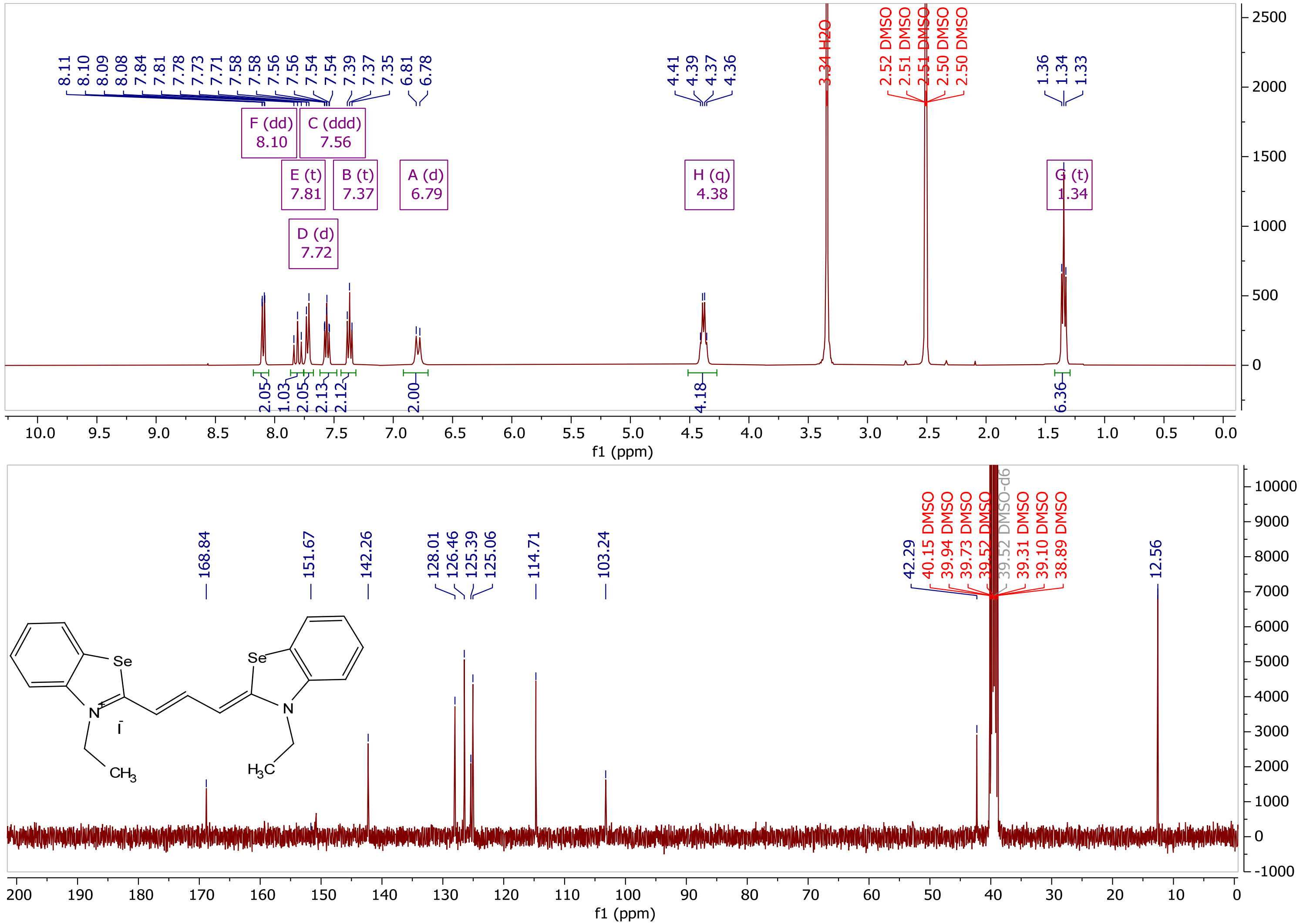

Figure S18 – ^{1}H and ^{13}C NMR spectra of trimethine cyanine dye **16**.

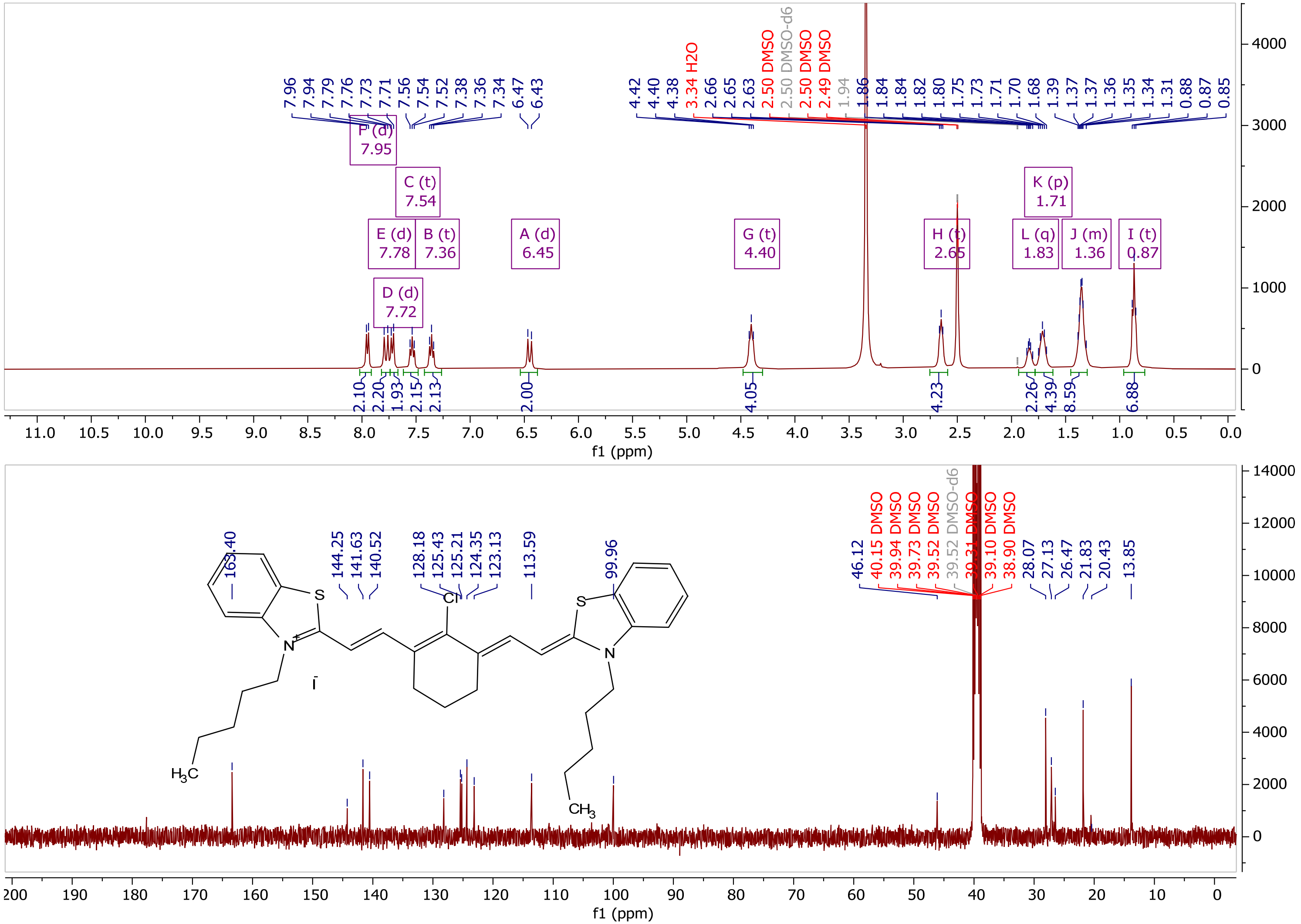

Figure S19 – ^{1}H and ^{13}C NMR spectra of heptamethine cyanine dye **17**.

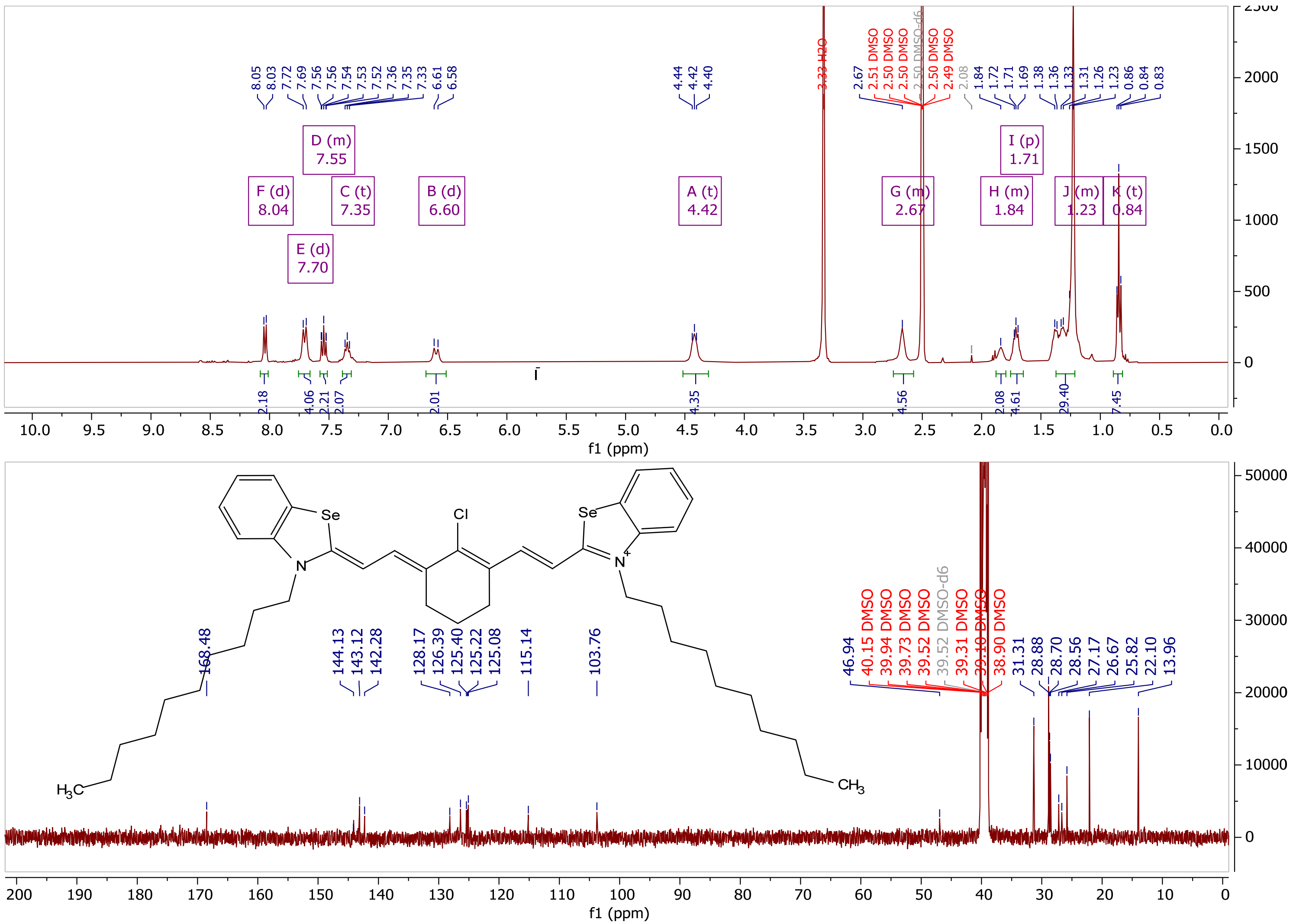

Figure S20 – ^{1}H and ^{13}C NMR spectra of heptamethine cyanine dye **18**.

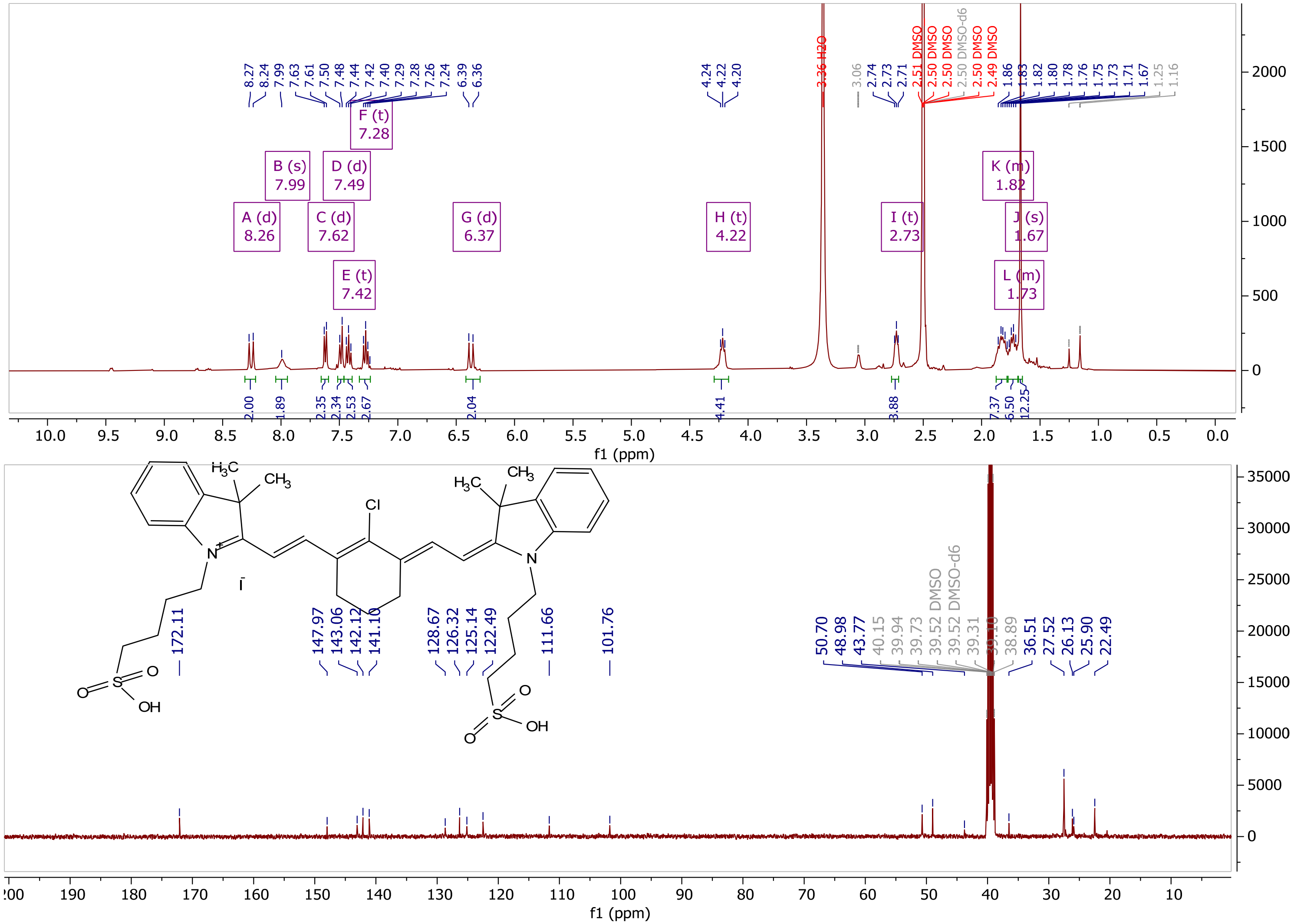

Figure S21 – ^{1}H and ^{13}C NMR spectra of heptamethine cyanine dye **19**.

SERVICIO DE ANÁLISIS ELEMENTAL, CROMATOGRAFÍA Y MASAS
C/ Espejo nº2 Edificio Multiusos I+D+i
Salamanca 37007

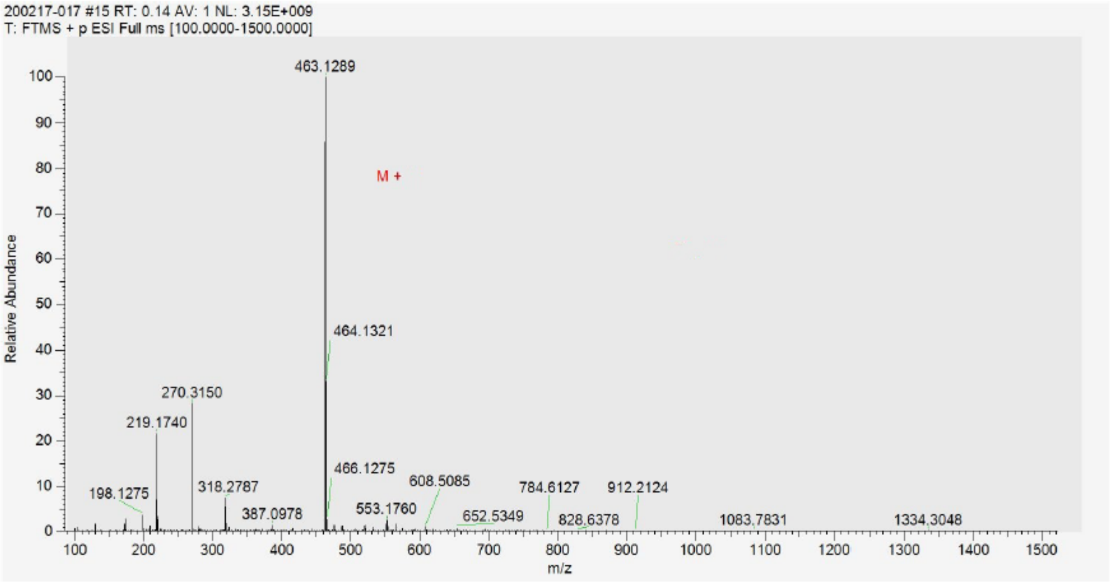

Fdo.: César Raposo Funcia
Director Técnico

Los resultados del análisis se refieren exclusivamente a la muestra sometida a ensayo

Figure S22 – ESI-HRMS spectrum of monomethine cyanine dye **8**.

SERVICIO DE ANÁLISIS ELEMENTAL, CROMATOGRAFÍA Y MASAS
C/ Espejo nº2 Edificio Multiusos I+D+i
Salamanca 37007

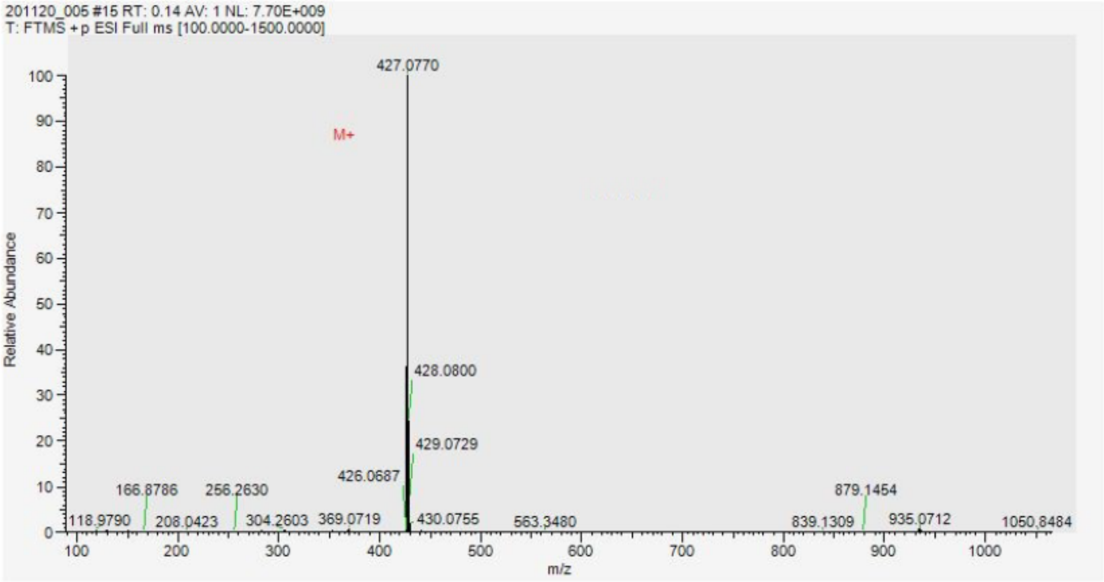

Fdo.: César Raposo Funcia
Director Técnico

Los resultados del análisis se refieren exclusivamente a la muestra sometida a ensayo

Figure S23 – ESI-HRMS spectrum of monomethine cyanine dye **9**.

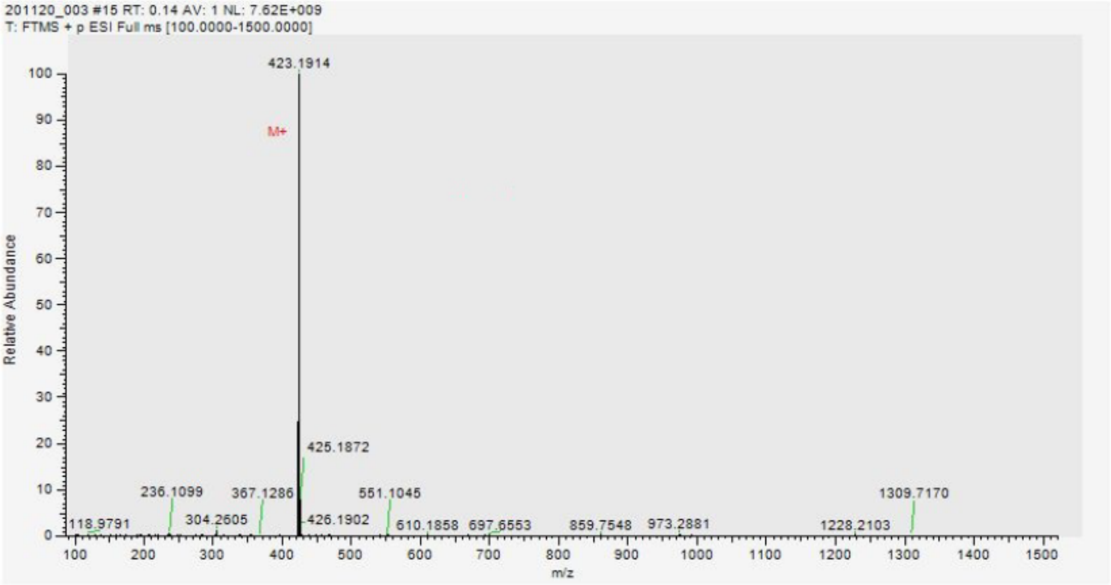

Fdo.: César Raposo Funcia
Director Técnico

Los resultados del análisis se refieren exclusivamente a la muestra sometida a ensayo

Figure S24 – ESI-HRMS spectrum of monomethine cyanine dye **10**.

SERVICIO DE ANÁLISIS ELEMENTAL, CROMATOGRAFÍA Y MASAS
C/ Espejo nº2 Edificio Multiusos I+D+i
Salamanca 37007

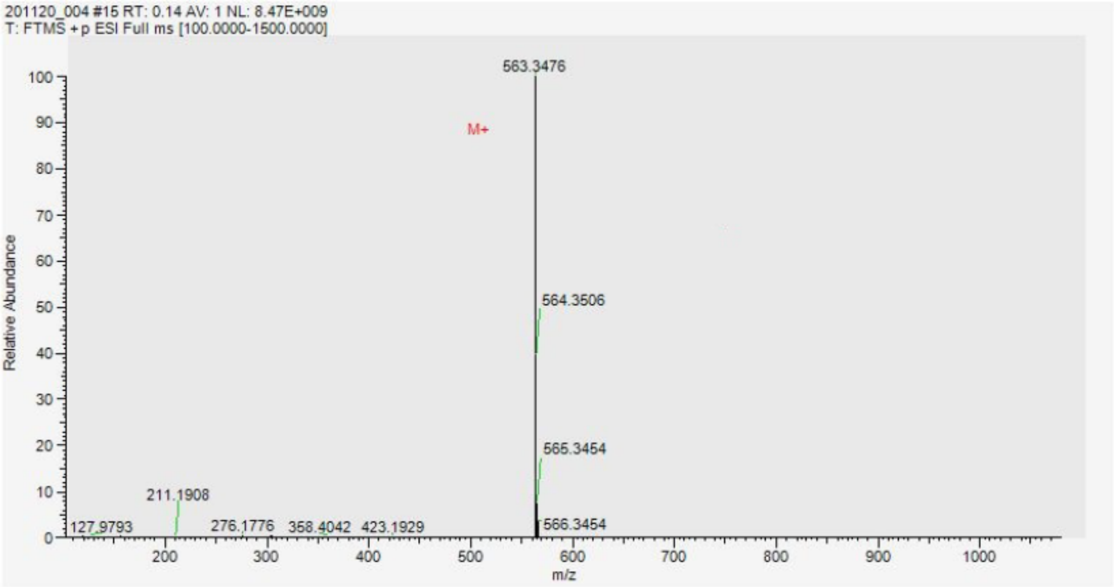

Fdo.: César Raposo Funcia
Director Técnico

Los resultados del análisis se refieren exclusivamente a la muestra sometida a ensayo

Figure S25 – ESI-HRMS spectrum of monomethine cyanine dye **11**.

SERVICIO DE ANÁLISIS ELEMENTAL, CROMATOGRAFÍA Y MASAS
C/ Espejo nº2 Edificio Multiusos I+D+i
Salamanca 37007

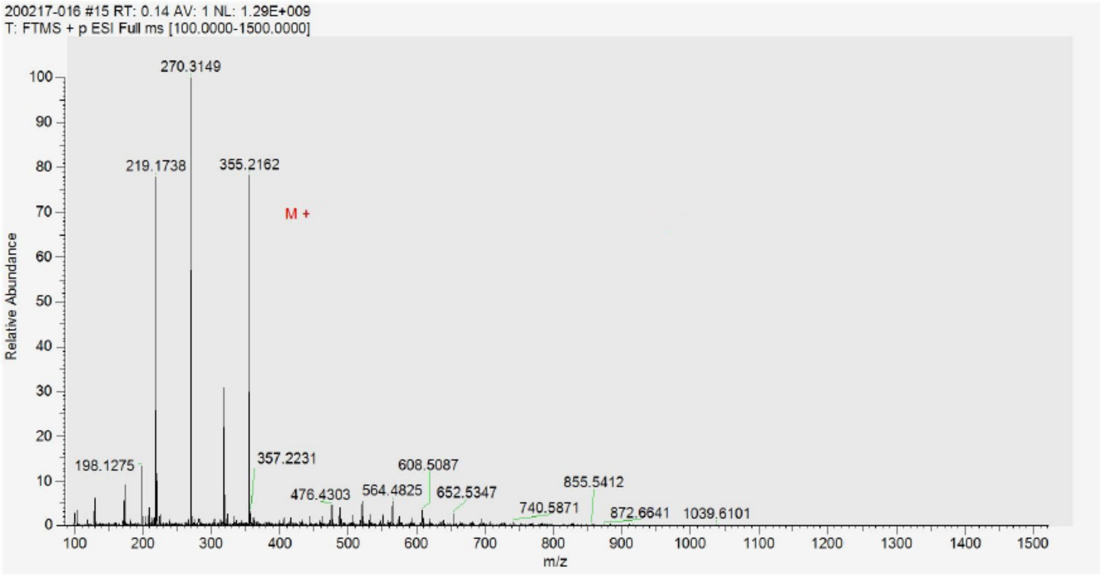

Fdo.: César Raposo Funcia
Director Técnico

Los resultados del análisis se refieren exclusivamente a la muestra sometida a ensayo

Figure S26 – ESI-HRMS spectrum of monomethine cyanine dye **14**.
